# Albumin versus Other Fluids for Fluid Resuscitation in Patients with Sepsis: A Meta-Analysis

**DOI:** 10.1371/journal.pone.0114666

**Published:** 2014-12-04

**Authors:** Libing Jiang, Shouyin Jiang, Mao Zhang, Zhongjun Zheng, Yuefeng Ma

**Affiliations:** Department of Emergency Medicine, Second Affiliated Hospital, School of Medicine & Institute of Emergency Medicine, Zhejiang University, Hangzhou, China; University of Dundee, United Kingdom

## Abstract

**Background:**

Early fluid resuscitation is vital to patients with sepsis. However, the choice of fluid has been a hot topic of discussion. The objective of this study was to evaluate whether the use of albumin-containing fluids for resuscitation in patients with sepsis was associated with a decreased mortality rate.

**Methods:**

We systematically searched PubMed, EMBASE and Cochrane library for eligible randomized controlled trials (RCTs) up to March 2014. The selection of eligible studies, assessment of methodological quality, and extraction of all relevant data were conducted by two authors independently.

**Results:**

In total, 15 RCTs were eligible for analysis. After pooling the data, we found there was no significant effect of albumin-containing fluids on mortality in patients with sepsis of any severity (RR: 0.94, 95% CI: 0.87, 1.02 and RD: –0.01, 95% CI: –0.03, 0.01). The results were robust to subgroup analyses, sensitivity analyses and trial sequential analyses.

**Conclusion:**

The present meta-analysis did not demonstrate significant advantage of using albumin-containing fluids for resuscitation in patients with sepsis of any severity. Given the cost-effectiveness of using albumin, crystalloids should be the first choice for fluid resuscitation in septic patients.

## Introduction

Sepsis is a common serious health problem. It is estimated that the annual number of patients with severe sepsis exceeds 750,000 in the United States and 19 million worldwide, with a short-term mortality of 20% to 30%, reaching up to 50% when shock is present [1]–[4]. Meanwhile, the total number of deaths from sepsis continues to increase [1]. It has been reported that sepsis is the leading cause of death among hospitalized patients in non-coronary intensive care units [5], [6].

Early fluid resuscitation is one of the key interventions for patients with sepsis which has been widely accepted by clinicians. However, the optimal choice of fluid remains inconclusive [7]–[10]. Albumin has been used as one type of resuscitation fluids since the Second World War [11]. However, until recently, the pragmatic value of albumin in sepsis is still under debate [12]–[15]. In 2011, a large meta-analysis which included 17 studies demonstrated that albumin use in patients with sepsis was associated with a decrease in mortality [16]. However, this study has some flaws. Firstly, it is vulnerable to bias because the most influential trial included was the pre-defined subgroup of patients with severe sepsis in the SAFE (the saline versus albumin fluid evaluation) study [13]. Secondly, six studies by Dr. Joachim Boldt (whose studies are suspected of lacking of integrity) were included in this meta-analysis [9]. As several large studies regarding which fluid should be used for resuscitation have been published recently, the purpose of this study was to further evaluate whether the use of albumin-containing fluids was associated with a decreased mortality rate in patients with sepsis.

## Methods

This meta-analysis was performed according to the Preferred Reporting Items for Systematic Reviews and Meta-Analyses (PRISMA) statement (Checklist S1) [17].

### Eligibility Criteria

Patients: patients with sepsis of any severity (including sepsis, severe sepsis and septic shock).

Intervention: fluid resuscitation.

Comparison: fluid resuscitation with albumin-containing fluids (of any concentration) vs. other resuscitation fluids (including any colloid or crystalloid).

Outcome: all-cause mortality at the longest follow-up available (including 48 h mortality, ICU mortality, hospital mortality, 28/30 days mortality, 90 days mortality, whichever was longest.).

Study Design: randomized controlled trials (RCTs).

### Literature Search and Study Selection

We systematically searched PubMed, EMBASE and Cochrane library up to March 2014. The following free text words or Medical Subject Headings were used: sepsis, septic, systemic inflammatory response syndrome, SIRS, septicemia, fluid therapy, resuscitation, plasma substitute, albumin and serum albumin. In addition, we also screened reference lists of all eligible studies and relevant reviews to obtain additional trials. There was no language restriction. The search strategy is showed in Text S2. Two investigators (JLB and MYF) independently screened the titles and abstracts of all records identified from the literature search. After excluding obviously non-relevant publications, potentially eligible articles were further screened in full text according to our pre-defined inclusion criteria. Discrepancies were resolved by consensus.

### Data Extraction and Quality Assessment

Data on the following items were extracted from the eligible studies by two investigators (LBJ AND MYF) independently: characteristics of studies, characteristics of patients, interventions and outcomes. Two reviewers (LBJ and MZ) independently and in duplicate assessed the methodological quality of each study by applying the following items: randomization-sequence generation, allocation concealment, blinding, intention-to-treat analysis, selective outcome reporting and the number of patients lost to follow-up. Randomization-sequence generation was considered adequate when the study described the method to generate the randomization sequence (such as computer-generated random numbers or random number table). Allocation concealment was considered adequate if researchers screening patients could not predict the next treatment for a patient. Blinding was considered adequate if both patients and investigators did not know which treatment the patients received. There was no evidence of selective outcome reporting if all stated endpoints were reported on and presented. Completeness of outcome data for each outcome was considered adequate if intention-to-treat analysis was performed and the lost follow-up rate should be within 10% [18].

### Statistical Analysis

Pooled risk ratios (RRs) with 95% confidence intervals (CIs) for all-cause mortality were calculated with RevMan 5.2.10 (http://tech.cochrane.org/revman/download) and STATA 12.0 (SERIAL NO. 40120519635). Heterogeneity between studies was measured by chi^2^ statistic (p<0.1) and quantified with I^2^ statistic [19]. If the I^2^ value was less than 50%, the fixed effects model was used to pool studies; otherwise, the random effects model was used. Several predefined subgroup analyses were performed according to patient's age (adult or pediatrics), type of resuscitation fluid in the control group (crystalloid or gelofusine or starch), concentration of albumin (4–5% solution or 20–25% solution), follow-up interval (ICU mortality, hospital mortality, 28/30 days mortality and 90 days mortality), disease severity (sepsis, severe sepsis and septic shock), and definition of sepsis (American College of Chest Physicians/Society of Critical Care Medicine, ACCP/SCCM, criteria or other criteria). Given the ALBIOS study was not limited to the resuscitation phase but included albumin supplementation for 28 days after enrollment, another subgroup analysis was conducted by the time interval between patients enrollment and randomization in the ALBIOS study (<6 h or 6–24 h) [7]. Meanwhile, we conducted sensitivity analyses and verified the robustness of our results by excluding either or both of the following studies: the EARSS study, which has not yet been published [20]; and ALBIOS study, in which albumin was used mainly for maintaining the serum albumin concentration of >30 g/L (not merely volume expansion) [21]. In addition, studies [22]–[30] with small sample sizes (<100 patients), studies with large sample sizes (>100 patients) [13], [20], [21], [31], [36], [37] and studies [26], [30], [31] on malaria which has a pathophysiology with many features in common with sepsis [32] were excluded to confirm the robustness of our results. Both random and fixed effects models were used. The sample size of a meaningful meta-analysis should be at least as large as a powered RCT. And updated meta-analyses of studies are vulnerable to random errors due to sparse data and repetitive testing of accumulated data [33]. Therefore, we conducted trial sequential analysis (TSA) to calculate the optimal required information size [34] (meta-analysis sample size) for our meta-analysis based on a baseline mortality rate of 31.7% in the control group which was calculated according to the 3 largest trials [13], [20], [21], a relative risk reduction of 10% [20], [21], 80% of power, and a type I error of 5%. We constructed monitoring boundaries to determine whether clinical trials could be terminated early when a p value is small enough to detect the expected effect. TSA was performed in TSA V.0.9 β (http://www.ctu.dk/tsa/). Publication bias was assessed by funnel plots and Egger’s test [35].

## Results

### Search Results and Study Characteristics

**Figure 1:**
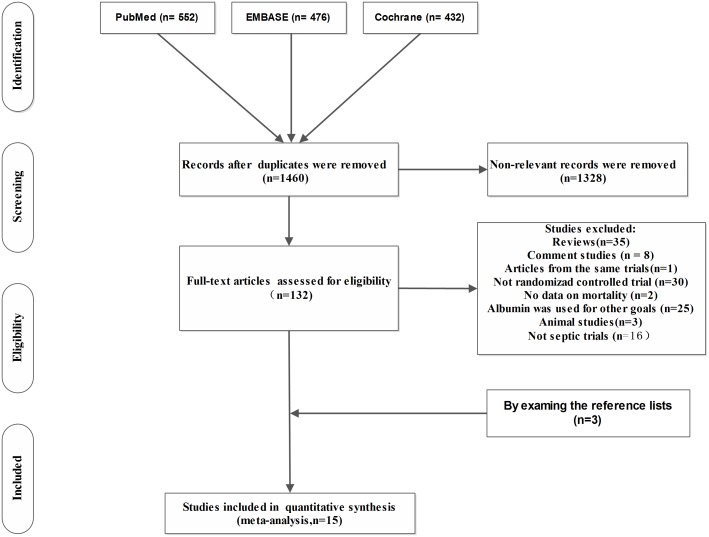
Flow chart for study selection.

A total of 1460 articles were identified through the literature search. According to our predefined inclusion criteria, 15 studies were included finally (Figure 1). Data on mortality were available in the published papers of all [13], [21], [23]–[28], [30], [31], [36], [37] but three of trials [20], [22], [29]; data for these were extracted from Delaney et al’s analysis [16] and Zheng yam et al’s analysis [38]. A total of 6998 septic patients were analyzed. Of these, 3225 patients received the albumin-containing fluids for resuscitation. The characteristics of all included studies are showed in Table 1.

**Table 1:** Characteristics of included studies.

Reference	Year	Adult/Children	Patient	No.of cases	Albumintype (s)	Controlfluid (s)	Resuscitationgoal (s)	Albuminvolume (ml)	Duration offollow-up
Rackowet al [23]	1983	Adult	Septic/hypovolaemic	26	5%	0.9% saline	PCWP≥15	2833	Hospital
			shock			6% HES			
Metildiet al [25]	1984	Adult	Severepulmonary	46	5%	Ringers lactate	Maintenanceof	9400	Hospital
			insufficiency				normal BE, Ph,SvO2		
Rackowet al [24]	1989	Adult	Severesepsis	20	5%	10% Pentastarch	PCWP≥15	975	Hospital
SAFE [13]	2004	Adult	Patientsin ICU	6997	4%	0.9% saline	The discretionof the	2376, first 3 days	28 days,
			requiringfluid				treatingclinicians		ICU
			resuscitation						Hospital
Veneman et al [29]	2004	Adult	Sepsisand post	63	20%	0.9% saline	MAP>70 mmHg	900	30 days
			surgicalpatients			10% HES	CVP 5–10 mmHg		
			with SIRS						
Maitlandet al [30]	2005	Children	Severe malaria,	61	4.5%	0.9% saline	20 mL/kg	20 mL/kg	ICU
			anaemia,severe						
			acidosis,respiratory						
			distress						
Maitlandet al [31]	2005	Children	Severe malariaand	150	4.5%	0.9% saline	To avoid	Moderate acidosis:	ICU
			metabolicacidosis				hypotension,	45 mL/kg	
							sustainedoliguria,	Severe acidosis:	
							worsening		
							metabolicacidosis	63 mL/kg	
Akechet al [26]	2006	Children	Severefalciparum	88	4.5%	Gelofusine	Resolution of	Moderate acidosis:	ICU
			malaria,metabolic				shock	46 mL/kg	
			acidosis,shock					Severe acidosis:	
								50 mL/kg	
Friedmanet al [22]	2008	Adult	sepsis andsuspected	42	4%	6% HES	Fixed volume 400 mL	400	Hospital
			hypovolemia			10% HES			
Van deret al [27]	2009	Adult	Septic andnon-septic	48	5%	0.9% saline	According tofluid	1500	ICU
			patients withor at risk			4% gelatin	challengeprotocol		
			for ALI/ARDS			6% HES			
Doleceket al [28]	2009	Adult	severesepsis	56	20%	6% HES	Intrathoracicblood volume	600	28 days
							index 850 mL/m2,		
							cardiac index		
							3.5 l/min/m2		
FEAST [36]	2011	Children	Severefebrile illness	3141	5%	0.9% saline	Resolution of	40 mL/kg, first 8 h	48 h,
			and impairedperfusion				impairedperfusion		28 days
EARSS [20]	2011	Adult	Septic shock	792	20%	0.9% NaCl	Fixed volume 100 mL	Fixed volume 100 mL,	28 days
							Every 8 h for3 days	every 8 h for 3 days	
CRISTAL [37]	2013	Adult	Patientsin ICU	2857	4%/5%or	Isotonic saline	The discretionof the	Not reported	28 days,
			requiringfluid		20%/25%		investigators		90 days
			resuscitation						
ALBIOS [21]	2013	Adult	Severesepsis or	1810	20%	Crystalloid	300 mL/dayuntil day	300 mL/day until day	28 days,
			septicShock			solution	28 or ICUdischarge	28 or ICU discharge	90 days

PCWP: Pulmonary capillary wedge pressure; MAP: Mean arterial pressure; CVP: Central venous pressure.

ALI: Acute lung injury; ARDS: Acute respiratory distress syndrome.

ICU: Intensive care unit.

### Quality Assessment

The methodical quality of all included studies was summarized in Table 2.

**Table 2:** Qualitative assessment of included studies.

Reference	Randomisation	Allocationconcealment	Blinding	Intentionto TreatAnalysis	Loss tofollow-up
Rackow et al [23]	Low risk	Unclear risk^d^	High risk	Low risk	Low risk
Metildi et al [25]	Low risk	Unclear risk^d^	High risk	Low risk	Low risk
Rackow et al [24]	Unclear risk^a^	Unclear risk^d^	High risk	Low risk	Low risk
SAFE [13]	Low risk	Low risk	Low risk	Low risk	Low risk
Veneman et al [29]	Unclear risk^a^	Low risk	High risk	Low risk	Low risk
Maitland et al [30]	Unclear risk^a^	Low risk	High risk	Low risk	Low risk
Maitland et al [31]	Unclear risk^a^	Low risk	High risk	Low risk	Low risk
Akech et al [26]	High risk^b^	Unclear risk^d^	High risk	Low risk	Low risk
Friedman et al [22]	Unclear risk^a^	Low risk	High risk	Low risk	High risk^c^
van der et al [27]	Unclear risk^a^	Low risk	High risk	Low risk	Low risk
Dolecek et al [28]	Low risk	Unclear risk^d^	High risk	Low risk	Low risk
FEAST [36]	Low risk	Low risk	High risk	Low risk	Low risk
EARSS [20]	Unclear risk^e^	Unclear risk^e^	High risk	Unclear risk^e^	Unclear risk^e^
CRISTAL [37]	Low risk	Low risk	High risk	Low risk	Low risk
ALBIOS [21]	Low risk	Low risk	High risk	Low risk	Low risk

a Just mention the word of random.

b A quasi-randomised design was used, whereby fluid interventions were allocated sequentially in blocks of ten.

c 4 patients (11%) were excluded because of inadequate data collection.

d not reported.

e The research has not yet been published.

### Mortality

**Figure 2:**
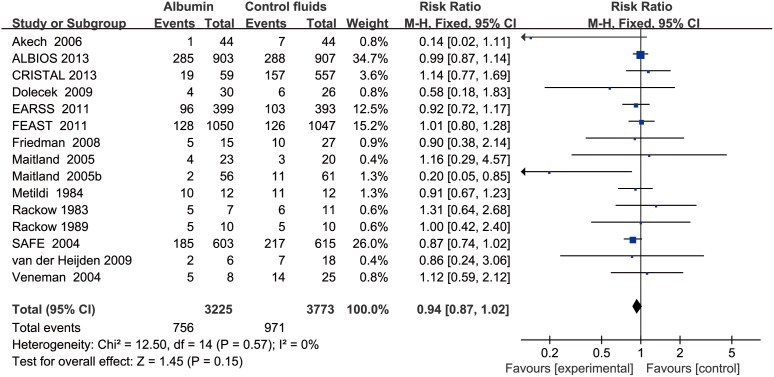
Forest plot showing the effects of albumin-containing fluids on all-cause mortality in patients with sepsis.

**Figure 3:**
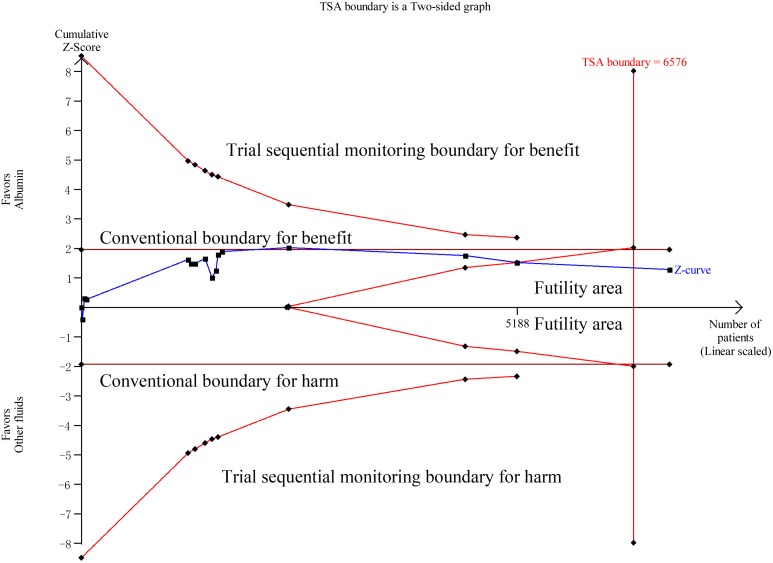
Trial sequential analysis of all-cause mortality in patients with sepsis. Trial sequential analyses assessing the effect of albumin on all-cause mortality in 15 studies. The diversity-adjusted required information size (6576 participants) was based on a relative risk reduction of 10%; an alpha of 5%; a beta of 2% and an event proportion of 31.7% in the control arm. The blue cumulative z curve was constructed using a random effects model.

**Figure 4:**
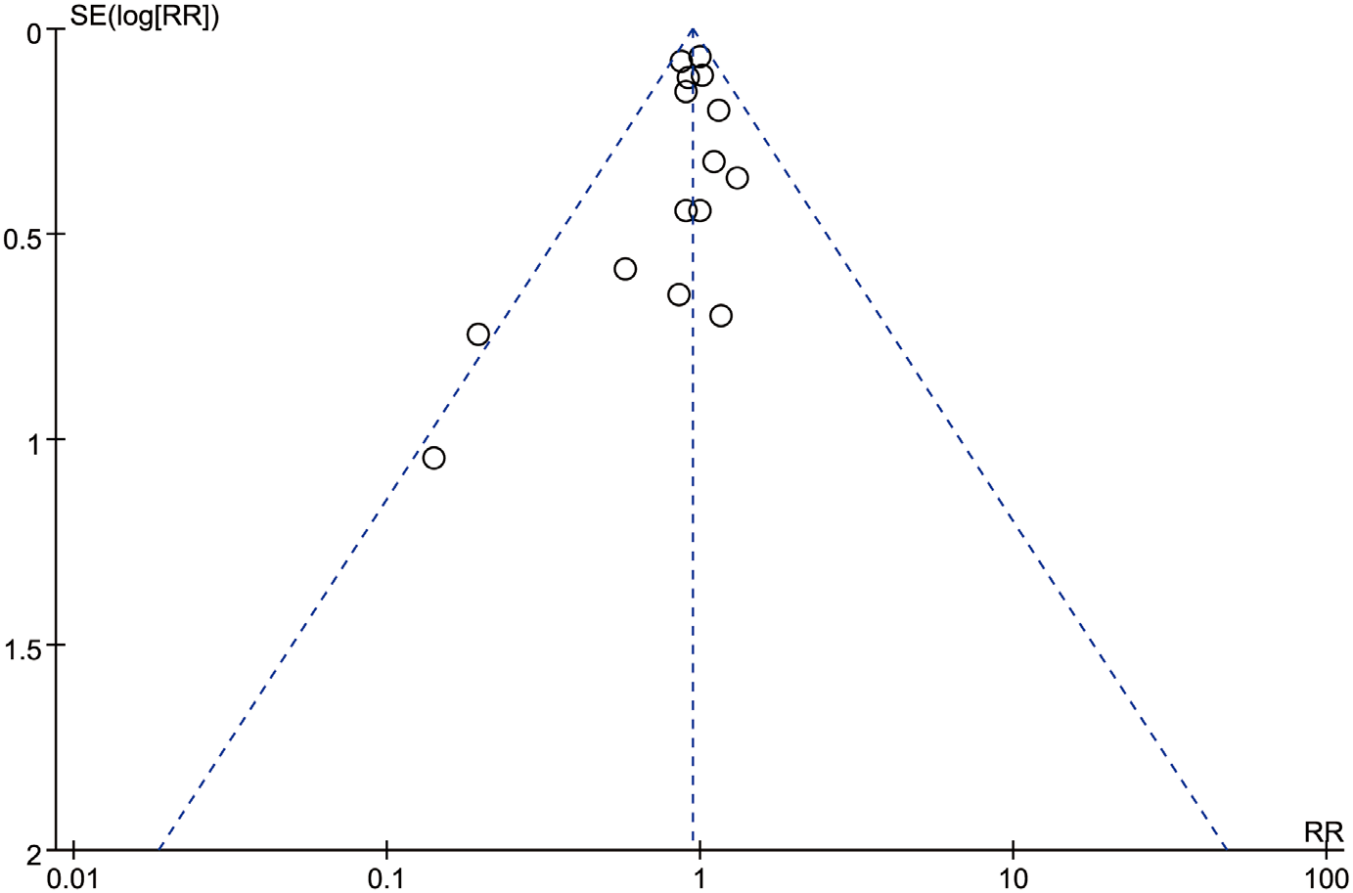
Funnel plot showing no significant publication bias.

Data on all-cause mortality were available from 15 RCTs [13], [20]–[29], [30], [31], [36], [37]. Although 90-day mortalities were reported in two studies, mortalities at 28 days which were the primary endpoints in these two studies were used to calculate the overall pooled RR for mortality [21], [37]. The results indicated that there was no effect of albumin on all-cause mortality in the fixed-effects model (RR: 0.94, 95% CI: 0.87, 1.02; p = 0.15) (Figure 2) or random-effects model (RR: 0.95, 95% CI: 0.88, 1.03; p = 0.20), with no heterogeneity between studies (I^2^ = 0%, p = 0.56). Trial sequential adjusted 95% CI of RR was 0.85 to 1.04 in the fixed effects model, and 0.86 to 1.04 in the random effects model. TSA showed that the diversity adjusted information size was 6576 which was less than that in our study (n = 6998) and the cumulative Z-curve surpassed the futility boundary, but it did not cross the trial sequential monitoring boundary for benefit or harm, indicating further studies are not required as they can unlikely change the current conclusion (whether benefit or harm) (Figure 3). The shape of the funnel plot and results of Egger’s test (p = 0.264) suggested no publication bias (Figure 4).

### Subgroup Analysis

**Figure 5:**
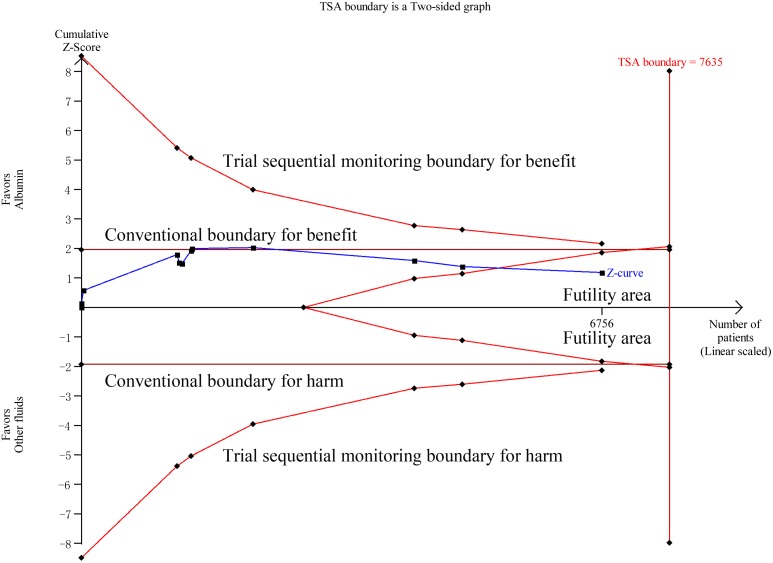
Trial sequential analysis of all-cause mortality in patients with sepsis comparing albumin with crystalloid solutions. Trial sequential analyses assessing the effect of albumin on all-cause mortality in 11 studies. The diversity-adjusted required information size (7635 participants) was based on a relative risk reduction of 10%; an alpha of 5%; a beta of 2% and an event proportion of 31.7% in the control arm. The blue cumulative z curve was constructed using a random effects model.

**Figure 6:**
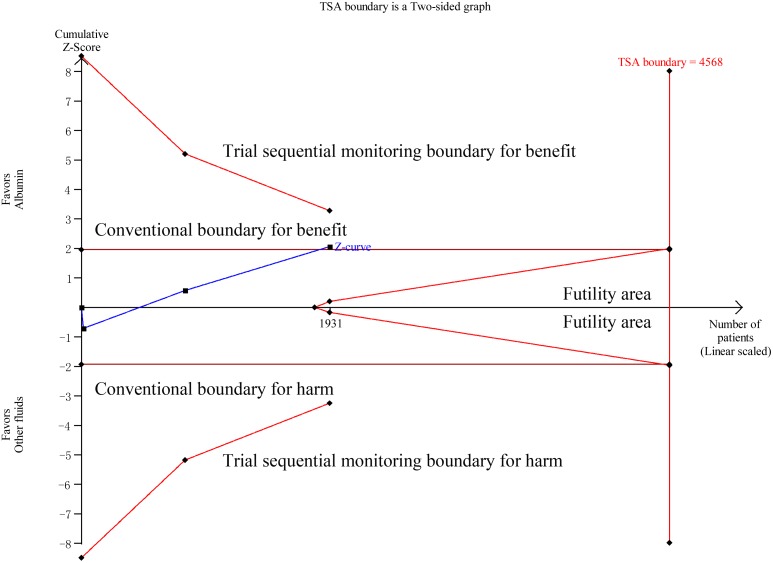
Trial sequential analysis of all-cause mortality in patients with septic shock. Trial sequential analyses assessing the effect of albumin on all-cause mortality in 3 studies. The diversity-adjusted required information size (4586 participants) was based on a relative risk reduction of 10%; an alpha of 5%; a beta of 2% and an event proportion of 40.3% in the control arm. The blue cumulative z curve was constructed using a random effects model.

In order to further evaluate the effect of albumin-containing fluids on all-cause mortality in patients with sepsis, several subgroup analyses were performed according to patient's age (adult or pediatrics), type of resuscitation fluid in the control group (crystalloid or gelofusine or starch), concentration of albumin (4–5% solution or 20–25% solution), follow-up interval (ICU mortality, hospital mortality, 28/30 days mortality and 90 days mortality), disease severity (sepsis, severe sepsis and septic shock), and definition of sepsis (American College of Chest Physicians/Society of Critical Care Medicine, ACCP/SCCM, criteria or other criteria). Given the ALBIOS study was not limited to the resuscitation phase but included albumin supplementation for 28 days after enrollment, another subgroup analysis was conducted by the time interval between patients enrollment and randomization in the ALBIOS study (<6 h or 6–24 h) [21]. As illustrated in Table 3, our results suggested that there was no significant effect of albumin on all-cause mortality in both adult and pediatric patients with sepsis. Eleven studies compared albumin with crystalloid [13], [20], [21], [23], [25], [27], [29], [30], [31], [36], [37]. Albumin was not associated with a significant reduction in all-cause mortality when compared to crystalloid (RR: 0.95, 95% CI: 0.87, 1.04; p = 0.25). Trial sequential adjusted 95% CI of RR was 0.87 to 1.04 in the fixed effects model, and 0.86 to 1.05 in the random effects model. The required information size was 7635 and the cumulative Z-curve crossed the boundary of futility, but it did not cross the trial sequential monitoring boundary for benefit or harm, indicating further studies are unlikely to change the current conclusion (Figure 5). Six studies [22]–[24], [27]–[29] compared albumin with hydroxyethyl starch and two studies [26], [27] compared albumin with gelofusine. The results indicated there is no evidence that albumin reduces mortality when compared with hydroxyethyl starch or gelofusine. And given the small sample sizes of these studies, we were unable to perform TSA. Although the difference of mortality between albumin group (both 4%–5% and 20%–25%) and control group did not reach statistical significance, we found 4%–5% albumin may be relative safer than 20%–25% albumin for fluid resuscitation (Table 3). In addition, we found albumin did not reduce all-cause mortality regardless of the follow-up time point. And these results were also not affected by the sepsis definition (5 studies [13], [20], [21], [28], [37] fitted the ACCP/SCCM criteria). Finally, a subgroup analysis was performed based on the disease severity. Albumin was associated with a small reduction in all-cause mortality when compared to an alternative resuscitation fluid in patients with septic shock (RR: 0.89, 95% CI: 0.80, 0.99; p = 0.04) [20], [21], [23]; however, this mortality benefit became insignificant when the comparison was limited to patients with sepsis [22], [37] and severe sepsis [13], [21], [24], [25], [27]–[29]. Moreover, this mortality benefit in patients with septic shock was not robust to TSA. Trial sequential adjusted 95% CI of RR was 0.74 to 1.07 both in the fixed and random effects model. Although the cumulative Z curve crossed conventional monitoring boundary for benefit, it did not cross the trial sequential monitoring boundary (Figure 6), suggesting that there was insufficient evidence to show a 10% reduction of all-cause mortality for 80% power, an α of 0.05, and a mortality rate of 40.3% in the control group. Given the potential bias of our study, further studies are needed to confirm whether albumin has an impact on mortality of patients with septic shock.

**Table 3:** Subgroup analyses.

Subgroup	No. ofStudies	Patients	FixedRR(95%CI)	RandomRR (95%CI)	I^2^	FixedRD (95%CI)	RandomRD (95%CI)	I^2^
**Total**	15	6983	0.94(0.87 to 1.02)	0.95(0.88 to 1.03)	0%	−0.01(−0.03 to 0.01)	−0.02(−0.04 to 0.00)	9%
<6 h	15	5757	0.93(0.85 to 1.02)	0.94(0.86 to 1.03)	0%	−0.02(−0.04 to 0.00)	−0.02(−0.05 to 0.00)	7%
6–24 h	15	6400	0.91(0.84 to 0.99)	0.92(0.85 to 1.00)	0%	−0.02(−0.04 to 0.00)	−0.03(−0.05 to 0.00)	8%
**Age of** **patients**								
Children	4	2345	0.92(0.74 to 1.14)	0.55(0.21 to 1.45)	63%	−0.01(−0.04 to 0.02)	−0.07(−0.16 to 0.03)	73%
Adults	11	4638	0.95(0.87 to 1.03)	0.95(0.87 to 1.03)	0%	–0.02(–0.05 to 0.01)	–0.02(–0.05 to 0.01)	0%
<6 h	11	3412	0.93(0.84 to 1.03)	0.94(0.85 to 1.03)	0%	–0.02(–0.06 to 0.01)	–0.02(–0.06 to 0.01)	0%
6–24 h	11	4055	0.91(0.84 to 1.00)	0.92(0.84 to 1.00)	0%	–0.03(–0.06 to 0.00)	–0.03(–0.06 to 0.00)	0%
**Types of** **control fluids**								
Crystalloid	11	6741	0.95(0.88 to 1.04)	0.95(0.87 to 1.04)	5%	–0.01(–0.03 to 0.01)	–0.02(–0.05 to 0.02)	38%
<6 h	11	5515	0.94(0.86 to 1.03)	0.94(0.86 to 1.04)	3%	–0.01(–0.04 to 0.01)	–0.02(–0.06 to 0.02)	38%
6–24 h	11	6158	0.92(0.85 to 1.00)	0.92(0.85 to 1.00)	0%	–0.02(–0.04 to 0.00)	–0.03(–0.06 to 0.01)	40%
Gelofusine	2	100	0.33(0.10 to 1.12)	0.42(0.05 to 3.18)	59%	–0.12(–0.24 to 0.00)	–0.13(–0.24 to – 0.02)	0%
Starch	6	169	0.91(0.62 to 1.32)	0.93(0.65 to 1.33)	0%	–0.04(–0.17 to 0.10)	–0.05(–0.18 to 0.09)	0%
**Concentrations of albumin**								
4%–5%	10	3691	0.90(0.79 to 1.01)	0.91 (0.80 to 1.04)	7%	–0.02(–0.05 to 0.00)	–0.05(–0.09 to 0.00)	32%
20%–25%	4	2676	0.97(0.86 to 1.09)	0.98 (0.87 to 1.09)	0%	–0.01(–0.04 to 0.03)	–0.01(–0.04 to 0.02)	0%
<6 h	4	1450	0.96(0.82 to 1.11)	0.97(0.83 to 1.12)	0%	–0.01(–0.06 to 0.03)	–0.02(–0.06 to 0.03)	0%
6–24 h	4	2093	0.92(0.82 to 1.03)	0.92 (0.82 to 1.03)	0%	–0.03(–0.07 to 0.01)	–0.03(–0.07 to 0.01)	0%
**Endpoints**								
ICUmortality	6	3587	0.88(0.76 to 1.02)	0.86 (0.64 to 1.14)	40%	–0.02(–0.04 to 0.00)	–0.05(–0.10 to 0.00)	58%
Hospitalmortality	5	1322	0.88(0.76 to 1.02)	0.89 (0.78 to 1.02)	0%	–0.04 (–0.09 to 0.01)	–0.04(–0.09 to 0.01)	0%
28/30-daymortality*	7	6607	0.96(0.88 to 1.04)	0.96 (0.88 to 1.04)	0%	–0.01(–0.03 to 0.01)	–0.01(–0.03 to 0.01)	0%
90-daymortality	2	2397	0.95(0.86 to 1.06)	0.95(0.86 to 1.06)	0%	–0.02(–0.06 to 0.02)	–0.02(–0.06 to 0.02)	0%
<6 h	2	1185	0.01(0.85 to 1.21)	0.01 (0.85 to 1.21)	0%	–0.01(–0.06 to 0.07)	–0.01(–0.06 to 0.07)	0%
6–24 h	2	1828	0.94(0.83 to 1.06)	0.93 (0.83 to 1.05)	0%	–0.03(–0.08 to 0.02)	–0.03(–0.08 to 0.02)	0%
**The definition of sepsis**								
ACCP/SCCM	5	4477	0.94(0.86 to 1.03)	0.94(0.86 to 1.04)	0%	–0.02(–0.05 to 0.01)	–0.02(–0.05 to 0.01)	0%
<6 h	5	3251	0.93(0.83 to 1.03)	0.93 (0.84 to 1.03)	0%	–0.02(–0.06 to 0.01)	–0.03(–0.06 to 0.01)	0%
6–24 h	5	3894	0.91(0.83 to 1.00)	0.91 (0.83 to 1.00)	0%	–0.03(–0.06 to 0.00)	–0.03(–0.06 to 0.00)	0%
Non -ACCP/SCCM	10	2506	0.93(0.78 to 1.12)	0.96(0.81 to 1.14)	4%	–0.01(–0.04 to 0.02)	–0.05(–0.11 to 0.01)	27%
**Severity of** **disease**								
Sepsis	2	658	1.10(0.77 to 1.57)	1.10 (0.77 to 1.57)	0%	0.03(–0.09 to 0.14)	0.03(–0.09 to 0.14)	0%
Severesepsis	7	2035	0.95(0.84 to 1.07)	0.95 (0.85 to 1.07)	0%	–0.02(–0.06 to 0.02)	–0.02(–0.06 to 0.02)	0%
Septicshock	3	1931	0.89(0.80 to 0.99)	0.89 (0.80 to 0.99)	0%	–0.04(–0.09 to 0.00)	–0.04(–0.09 to 0.00)	0%

RR, Relative Risk; 95% CI, 95% Confidence Intervals; RD, Risk difference; Fixed, fixed - effects model; Random, random - effects model.

*, 28/30 – day mortality were not stratified according to the time interval between patient enrollment and randomization in the ALBIOS study.

ACCP/SCCM, American College of Chest Physicians/Society of Critical Care Medicine.

### Sensitivity analysis

Sensitivity analyses were performed by excluding the following studies successively: EARSS study [20], which was abstract from conference proceedings; ALBIOS study [21], in which the primary aim of albumin-containing fluids administration was not for initial resuscitation; small studies (<100 patients) [22]–[25], [26]–[31]; large studies (>100 patients) [13], [20], [21], [31], [36], [37]; and studies [26], [30], [31] on malaria which has a pathophysiology with many features in common with sepsis [39]. The results indicated that the exclusion of these studies did not change our primary outcomes (Table 4).

**Table 4:** Sensitivity analysis.

Excluding studies	No. ofPatients	RR(95%CI)Fixed	Random	I^2^	RD(95%CI)Fixed	Random	I^2^
EARSS[22]	6206	0.94(0.86 to 1.03)	0.95(0.88 to 1.04)	0%	–0.01(–0.04 to 0.01)	–0.02(–0.05 to 0.00)	16%
<6 h	4965	0.93(0.84 to 1.03)	0.94(0.86 to 1.04)	0%	–0.02(–0.04 to 0.01)	–0.03(–0.06 to 0.00)	14%
6–24 h	5638	0.91(0.84 to 1.00)	0.92(0.85 to 1.00)	0%	–0.02(–0.05 to 0.00)	–0.03(–0.06 to 0.00)	15%
ALBIOS [33]	5188	0.91(0.82 to 1.01)	0.93(0.84 to 1.02)	0%	–0.02(–0.04 to 0.00)	–0.03(–0.06 to 0.00)	13%
Both[22], [33]	4396	0.91(0.81 to 1.02)	0.93(0.83 to 1.03)	0%	–0.02(–0.04 to 0.00)	–0.04(–0.07 to 0.00)	19%
Smallstudies [22]–[30]	6650	0.95(0.87 to 1.03)	0.95(0.85 to 1.07)	31%	–0.01(–0.03 to 0.01)	–0.02(–0.05 to 0.01)	46%
<6 h	5409	0.94(0.85 to 1.03)	0.95(0.84 to 1.07)	29%	–0.02(–0.04 to 0.01)	–0.02(–0.06 to 0.01)	45%
6–24 h	6052	0.92(0.84 to 1.00)	0.93(0.83 to 1.03)	24%	–0.02(–0.04 to 0.00)	–0.03(–0.06 to 0.01)	48%
Large studies	348	0.85(0.64 to 1.13)	0.94(0.75 to 1.17)	0%	–0.05(–0.13 to 0.04)	–0.08(–0.15 to 0.00)	0%
[13, 20,21, 31, 36, 37]							
Trials onmalaria [26], [30], [31]	6750	0.96(0.88 to 1.04)	0.96(0.88 to 1.04)	0%	–0.01(–0.03 to 0.01)	–0.01(–0.03 to 0.01)	0%
<6 h	5508	0.95(0.86 to 1.04)	0.95(0.87 to 1.04)	0%	–0.01(–0.04 to 0.01)	–0.01(–0.03 to 0.01)	0%
6–24 h	6152	0.93(0.86 to 1.01)	0.93(0.86 to 1.01)	0%	–0.02(–0.04 to 0.00)	–0.01(–0.03 to 0.01)	0%

RR, Relative Risk; 95% CI, 95% Confidence Intervals; RD, Risk difference; Fixed, fixed - effects model; Random, random - effects model.

Both, EARSS+ALBIOS were excluded.

## Discussion

A total of 15 studies enrolling 6998 patients were eligible for evaluating the effect of albumin-containing fluids on all-cause mortality in patients with sepsis [13], [20]–[29], [30], [31], [36], [37]. The results of this meta-analysis indicated that the use of albumin-containing fluids for the resuscitation of patients with sepsis of any severity was not associated with lower death rates compared with other fluid resuscitation regimens.

Cardiovascular system can be impaired by sepsis which may be mediated by multiple mechanisms, with the result of tissue hypo-perfusion. Meanwhile, the increased intravascular space and capillary permeability which result from direct cell damage and the release of inflammatory mediators can further increase the amount of fluid required and thus complicate the resuscitative process [40]. Therefore, maintenance of adequate intravascular volume and tissue perfusion is critical with regard to patients’ outcome, and early adequate fluid resuscitation has been shown to improve the prognosis of septic patients [41]. Until now, the choice of fluid for resuscitation in patients with sepsis remains controversial. In recent years, several large RCTs and systematic reviews have reported that the use of hydroxyethyl starch, the commonly used colloid solution, is associated with a significant increased risk of acute kidney injury and death in critically ill patients [8]–[10]. Meanwhile, the results from the SAFE study and a subsequent Meta-analysis have shown that albumin as a resuscitation fluid for patients with sepsis may significantly reduce the risk of death [13], [16]. These conflicting results have raised a re-emerging debate regarding which fluid on earth should be used for fluid resuscitation in patients with sepsis.

Crystalloid solutions are widely used in fluid resuscitation of critically ill patients. In addition to their efficiency, crystalloids are popular also because they are readily available and cheap. However, the use of crystalloids is not without drawbacks. Because crystalloids are composed of only small particles such as sodium ions and chlorine ions, large infusion of crystalloids especially normal saline may result in hypernatronemia and hyperchloremic acidosis which have proven to be associated with coagulation derangements and renal, cerebral, gastrointestinal and respiratory dysfunction [40]. In addition, due to their lower molecular weight, crystalloid solutions can easily across the damaged semi-permeable membrane of capillaries which often results in a shorter intravascular persistence of fluids and may aggravate lung edema [42]. Human albumin is a natural protein which accounts for 50%–60% of all plasma proteins and nearly 80% of plasma colloid osmotic pressure [11]. Compared with crystalloids, albumin can efficiently hold intravenous fluids due to their larger molecular weight [35], [37]. Although there is the possibility that the increased membrane permeability can augment the extravasation of fluid into the interstitium due to leakage of albumin [31], it has been reported that the required amount of fluid to achieve the same resuscitation endpoint can be two to three times higher in the crystalloids group than in the colloids group [13], [14], [21]. As a natural colloid, human albumin is supported by the findings that septic patients receiving albumin-containing fluids usually have higher colloid osmotic pressure, central venous pressure, and slower heart rate than those who received crystalloids [21]–[23], [27], [28]. It's worth noting that whether patients are in septic or non-septic status, resuscitation with albumin showed greater cardiac responses than normal saline [43]. Unfortunately, until now, data regarding differences in the above mentioned indicators between albumin and artificial colloids were controversial [23]–[25], [27], [29], [43].

Human albumin also has multiple roles other than its oncotic properties: 1) transporting other biologically active molecules; 2) antioxidant; 3) anti-inflammatory action; 4) inhibition of platelet aggregation; 5) capacity for reducing capillary permeability and maintaining endothelial cell integrity; and 6) buffering the acid-base equilibrium [4], [16], [21]. It has been reported that hypoalbuminemia, which is common in critically ill patients (including septic patients), is associated with poor clinical outcomes [44]–[46]. Chou et al reported that for patients with severe sepsis due to secondary peritonitis, albumin administration may reduce 28-day mortality, however this mortality benefit was limited to patients whose baseline serum albumin is 20 g/L or lower [45].

The abnormal accumulation of fluid in the extravascular space of the lung along with severe inflammation may cause impairment of oxygenation and are strongly associated with a high risk of death [40], [47]. Therefore, whether albumin can be used as a resuscitation fluid has been comprehensively appraised by oxygenation, pulmonary edema, organ performance, and resource utilization. As mentioned above, due to its oncotic properties and non-oncotic properties, albumin may decrease the extravasation of fluid from vessels into interstitial spaces and thus reduce the degrees of pulmonary edema and improve oxygenation [40], [48]–[51]. A recent meta-analysis has demonstrated that the use of albumin is associated with improved oxygenation when compared to crystalloid solutions [47]. However, this effect was not observed in the study by Van der et al [27]. This inconsistent result may be partially explained by that 5% albumin was used in the study by Van der et al, whereas 25% albumin was used in the meta-analysis. Dolecek et al reported that 20% albumin could significantly reduce the amount of extravascular lung water when compared to 6% HES [28]. Nevertheless, oxygenation was not shown to be better in patients treated with 20% albumin [22], [28], [52]. In addition, there was no significant difference in pulmonary edema and oxygenation function between the 5% albumin group and the 6% HES group [24], [27]. As for organ function, patients in the albumin group, as compared with those in the crystalloid group, had a higher SOFA sub-scores for liver [13], [21]. It may be explained by the presence of bilirubin which was associated with the methods used to prepare albumin solutions [13], [21]. In addition, in the ALBIOS study, the authors also found a slightly higher SOFA sub-scores for coagulation in the albumin group, which was attributed to the dilution of the hemoglobin content due to early and large intravascular volume expansion [21]. Finally, most studies showed that there was no effect of albumin on the length of stay in ICU/hospital, duration of mechanical ventilation, requirement of renal replacement therapy [14], [20], [21].

Hitherto, there are still many unsolved issues about albumin administration in patients with sepsis. Firstly, timing of albumin administration. The optimal time to administer albumin to patients with sepsis has not yet been explored. However, it has been reported that fluid resuscitation improve microvascular perfusion in the early but not in the late phase of sepsis, and this effect is independent of the type of fluid [53]. Thus, it seemed that the timing of fluid resuscitation is more important than the type of fluid [54]. Secondly, concentration of albumin. In general, 4%–5% albumin is usually used for resuscitation and 20%–25% albumin is usually used for maintaining normal serum albumin levels. However, in a large meta-analysis, the authors reported that hyperoncotic albumin decreased the odds of acute kidney injury and death by 76% and 48%, respectively [55]. And hyperoncotic albumin seems to improve oxygenation better than hypooncotic albumin [47]. Moreover, the results of the SAFE study indicated that resuscitation with 4% albumin might increase mortality in patients with traumatic brain injury [13]. It is worth noting that the choice of albumin concentration may also depend on the type of fluid which is administrated simultaneously. In the present meta-analysis, we found 4%–5% albumin may be relative safer than 20%–25% albumin for fluid resuscitation. Thirdly, dose of albumin. Until now, no researches have yet been designed to assess the dose-response relationship between albumin exposure and mortality rate in patients with sepsis. Recent evidence have suggested that whether in the early or late phase of resuscitation, net positive fluid balance is associated with worse outcome [56], [57], [58]. In a large meta-analysis, the authors reported that albumin reduces morbidity in acutely ill hospitalized patients, however this effect was significantly influenced by the albumin dose in the control group [59]. Finally, the high cost of albumin may limit its wide applicability. Albumin can be anywhere between 20 and 100 times more expensive than crystalloids, therefore, the cost effectiveness of albumin should be incorporated into the stands of care. It has also been reported that the number of patients needed to treat (NNT) to avoid one additional death is 45, namely the cost per case avoided was $31,220, based on the results of the EARSS study [60], [61]. Therefore, if there is no significant advantage of albumin in reducing mortality rate, it is difficult to justify unrestricted use of albumin for resuscitation of patients with sepsis. Though our results indicated that further studies are unlikely to change the current conclusion but considering the above mentioned issues and potential bias, further studies are needed to confirm whether albumin has an impact on mortality of patients with sepsis.

### Strengths and Limitations of This Meta-Analysis

There are several strengths of this meta-analysis. First, the present meta-analysis was performed according to the Preferred Reporting Items for Systematic Reviews and Meta-Analyses (PRISMA) statement protocol [17]. Three electronic databases which are recommended by the Cochrane Collaboration were searched for relevant studies. The screening of eligible studies, assessment of methodological quality and data extraction were conducted independently and in duplicate. Second, we only included RCTs in this review to minimize potential bias and there were enough number of patients to address this question. Meanwhile, there was no significant heterogeneity among included studies. Third, several pre-defined subgroup analyses and sensitivity analyses were performed to verify the robustness of our results and trial sequential analysis was performed to eliminate random errors.

This study has several limitations. Firstly, although there was insignificant heterogeneity between studies in this meta-analysis, the methodological quality of all included studies was variable and all studies were open label except for the SAFE study [13]. Secondly, patients with sepsis in six studies were only a subgroup of the total populations studied. Thirdly, there is evidence that a longer observation period for mortality, such as 90 days, is appropriate to assess the real effects of treatments in critically ill patients [62], [63]. Unfortunately, in our meta-analysis, 90 days mortality was only reported in two studies [21], [37]. Another limitation of our meta-analysis is that there were relatively few studies comparing albumin and artificial colloids were included. As mentioned above, albumin was compared with hydroxyethyl starch in six studies [22]–[24], [27]–[29], and compared with gelofusine in two studies [26], [27]. As we all know, hydroxyethyl starch is associated with an increased risk of acute kidney injury and death [8]–[10], thus, indirectness is a major limitation for the comparison of albumin with hydroxyethyl starch and there is insufficient evidence to make any firm conclusions on comparisons of albumin with artificial colloids based on these sparse data. In addition, because the first research included in our study can be retrospect to 1983, the influence of existing standards of care on outcome may have affected the results of this study and different albumin manufacturers may also have an impact on the results.

## Conclusion

Although albumin has many theoretical advantages, these have not been supported by clinical trials. The present meta-analysis did not demonstrate significant advantages of albumin over other fluids for resuscitation in patients with sepsis of any severity. Given the tremendous economic burden of albumin, crystalloids should be the first choice for fluid resuscitation in septic patients.

## Supporting Information

Text S1 **Search strategy.**
(PDF)

Checklist S1 **The Preferred Reporting Items for Systematic Reviews and Meta-Analyses (PRISMA) statement.**
(DOC)
