# Supplementary material for: Albumin versus Other Fluids for Fluid Resuscitation in Patients with Sepsis: A Meta-Analysis
Source: PLoS One. 2014 Dec 4;9(12):e114666. doi: 10.1371/journal.pone.0114666 (PMC4256427; doi:10.1371/journal.pone.0114666)
Supplement: Text S1 — Search strategy. (PDF) [file pone.0114666.s001.pdf]

Search strategy

Recent queries in pubmed
Search,Query,Items found,Time
#26,"#25 AND #20 AND #9 Filters: Publication date to 2014/03/31",552,23:45:41
#25,"Search #23 OR #24,614056,23:45:03
#24,"Search ""Randomized Controlled Trials as Topic""[Mesh]",91098,23:44:08
#23,"Search ((""randomized controlled trial""[Title/Abstract]) OR random[Title/Abstract]) OR ""Randomized Controlled Trial"" [Publication Type]",535166,23:21:50
#22,"Search random[Title/Abstract]",173371,23:17:33
#21,"Search ""randomized controlled trial""[Title/Abstract]",29357,23:15:01
#20,"Search #10 OR #11 OR #12 OR #13 OR #14 OR #15 OR #16 OR #17 OR #18 OR #19,8115,23:08:28
#19,"Search ""Plasma Substitutes""[Mesh]",6144,23:07:25
#18,"Search ""Resuscitation""[Mesh]",72393,23:07:05
#17,"Search ""Fluid Therapy""[Mesh]",14355,23:06:44
#16,"Search ""Serum Albumin""[Mesh]",68518,23:06:16
#15,"Search "" serum albumin""",90105,23:06:05
#14,"Search ""Albumins""[Mesh]",137904,23:05:34
#13,"Search albumin",202460,23:04:31
#12,"Search ""fluid therapy""[Title/Abstract]",2206,23:02:52
#11,"Search ""plasma substitute""[Title/Abstract]",355,23:02:22
#10,"Search resuscitation[Title/Abstract]",38215,23:01:22
#9,"Search #1 OR #2 #3 OR #4 OR #5 OR #6 OR #7 OR #8,141545,22:54:15
#8,"Search ""Shock, Septic""[Mesh]",18050,22:52:47
#7,"Search ""Sepsis""[Mesh]",90349,22:52:10
#6,"Search ""Systemic Inflammatory Response Syndrome""[Mesh]",93248,22:51:30
#5,"Search ""sepsis syndrome""[Title/Abstract]",703,22:50:11
#4,"Search ""systemic inflammatory response syndrome""[Title/Abstract]",2982,22:47:02
#3,"Search septicemia[Title/Abstract]",10866,22:43:14
#2,"Search “septic shock” [Title/Abstract]",14305,22:42:42
#1,"Search sepsis[Title/Abstract]",64237,22:40:57

Embase
Session Results
....

No.	Query Results	Results
#35.	#33 AND #27 AND #15	476
#34.	#28 OR #29 OR #30 OR #31 OR #32 OR #33	583,153
#33.	'randomized controlled trial (topic)'/exp/mj	2,548
#32.	'randomized controlled trial'/exp/mj	7,961
#31.	'randomized controlled trial'/exp OR 'randomized controlled trial'	400,260
#30.	'randomized controlled trail'	71
#29.	'randomized controlled study'/exp OR 'randomized controlled study'	344,014
#28.	random	195,507
#27.	#16 OR #17 OR #18 OR #19 OR #20 OR #21 OR #22 OR #23 OR #24 OR #25 OR #26	279,934
#26.	'plasma substitute'/exp/mj	24,879

Search strategy

#25.	'fluid resuscitation'/exp/mj	988
#24.	'fluid therapy'/exp/mj	24,905
#23.	'intravenous administration'/exp/mj	625
#22.	'fluid therapy'/exp/mj	24,905
#21.	'albumin'/exp/mj	19,276
#20.	'serum albumin'/exp OR 'serum albumin'	85,171
#19.	'albumin'/exp OR albumin	185,719
#18.	'fluid therapy':ab,ti	2,777
#17.	'plasma substitute':ab,ti	379
#16.	resuscitation:ab,ti	48,977
#15.	#1 OR #2 OR #3 OR #4 OR #5 OR #6 OR #7 OR #8 OR #9 OR #10 OR #11 OR #12 OR #13 OR #14	143,755
#14.	'sepsis syndrome':ab,ti	851
#13.	syndrome:ab,ti	818,675
#12.	'sepsis'/exp OR sepsis AND syndrome:ab,ti	18,634
#11.	'systemic inflammatory response syndrome'/exp/mj	75,011
#10.	'septic shock'/exp/mj	14,478
#9.	'septic shock'/exp/mj	14,478
#8.	'sepsis'/exp/mj	73,214
#7.	'sepsis syndrome':ab,ti	851
#6.	'septic shock':ab,ti	19,724
#5.	'systemic inflammatory response syndrome':ab,ti	3,977
#4.	'systemic in-flammatory response syndrome':ab,ti	1
#3.	septicemia:ab,ti	12,650
#2.	sepsis:ab,ti	87,039
#1.	'sepsis'/exp OR sepsis	192,952

....
